# *Bacillus velezensis* EEAM 10B Strengthens Nutrient Metabolic Process in Black Soldier Fly Larvae (*Hermetia illucens*) *via* Changing Gut Microbiome and Metabolic Pathways

**DOI:** 10.3389/fnut.2022.880488

**Published:** 2022-05-19

**Authors:** Yaxin Pei, Sijie Zhao, Xiang Chen, Jiran Zhang, Hongyuhang Ni, Mengxiao Sun, Hui Lin, Xinyu Liu, Hongge Chen, Sen Yang

**Affiliations:** ^1^Department of Microbiology, School of Life Sciences, Henan Agricultural University, Key Laboratory of Agricultural Microbial Enzyme Engineering (Ministry of Agriculture), Zhengzhou, China; ^2^Department of Infectious Diseases and Public Health, Jockey Club College of Veterinary Medicine and Life Sciences, City University of Hong Kong, Kowloon, Hong Kong SAR, China

**Keywords:** intestinal probiotics, *Bacillus velezensis*, insect–microbiota interaction, microbial community, metabolome

## Abstract

Insects are a potential alternative protein source to solve the food shortage crisis. Previous studies have illustrated that probiotics can improve the substrate conversion efficiency of insects and increase insect protein content. However, the effects of probiotics on insect physiology and nutrient metabolism are still not well understood. Here, the black soldier fly larvae (BSFL), *Hermetia illucens* (Diptera: Stratiomyidae), was used as a study subject to deeply investigate the specific interaction among a novel probiotic, *Bacillus velezensis* EEAM 10B (10B), intestinal microbiota, and the host. In this study, the effects of 10B on the survival and physiology of BSFL were first analyzed. It shows that 10B significantly elevated the substrate conversion rate, average dry weight, and protein content of BSFL by 5%, 0.13 g/pc, and 8%, respectively. Then, we assessed the effect of 10B on the microbial community composition in the gut and frass of BSFL using Illumina Miseq sequencing. It shows that 10B significantly altered the microbial composition of the gut, but not that of the frass. Pearson’s correlation analysis further showed that the *Bacillus*, *unclassified_of_Caloramatoraceae*, and *Gracilibacillus* were positively correlated with the survival rate, crude protein content, and substrate conversion rate of BSFL. To further investigate the effect of 10B on host metabolism, metabolic analyses on germ-free BSFL, monobacterial intestinal BSFL, and natural BSFL were also performed. The results proved that 10B (i) played a vital role in the survival of BSFL; and (ii) regulated the amino acid synthetic and metabolic process of BSFL, thus leading to the rise of the protein content of BSFL. In addition, vitamin backfill assays verified that the BSFL survival rate was significantly improved by supplying the germ-free BSFL with riboflavin, which further suggests that 10B determines the survival of BSFL *via* delivering riboflavin. Overall, this study provides a reference for understanding the comprehensive contribution of a specific probiotic to its host.

## Introduction

The critical shortage of protein sources is currently a significant bottleneck for the development of the animal feed industry. This dilemma will be accentuated by the dramatic increase in the world population and the demand for meat in daily diets (1, 2). Therefore, finding other potential protein sources to replace expensive traditional sources has become an urgent need (3, 4). Insects are a promising and sustainable alternative protein source, and their applications as protein additives in animal feed have been the focus of recent studies (5, 6). The black soldier fly larvae (BSFL), *Hermetia illucens* (L.) (Diptera: Stratiomyidae), has become one of the chief insects used for bioconversion in the world (7), due to its capability to adapt to adverse environments (8), control pests and harmful bacteria (9), and convert various organic wastes into high-quality protein (10).

The gut microbiota is key to converting organic wastes in BSFL and thus determining their nutritional phenotypes (11). Therefore, more studies are concentrated on converting substrates into insect proteins during the conversion process. As an open ecological system, the insect intestine allows many external microorganisms to colonize in it and interact with the initial gut microbiota (12). The addition of probiotics to improve substrate utilization and conversion by BSFL is encouraging (13). Functional bacteria isolated from the BSFL gut have been shown to shorten the number of hatching days, increase the weight of prepupae and pupae, and improve the efficiency of substrate conversion (14). For instance, with the addition of *Bacillus subtilis* to the chicken manure, the larva weight, chicken manure reduction rate, and chicken manure conversion rate of BSFL was, respectively, increased by 15.9, 13.4, and 12.7% (15). In another study, BSFL fed with soybean curd residues and *Lactobacillus buchneri* had a significantly higher fat content (30.0 ± 0.6%), bioconversion rate (6.9 ± 0.3%), dry mass reduction (55.7 ± 0.9%), and crude protein content (55.3 ± 0.6%) than the control group (16). However, there are very few studies on the interactions among the probiotics, intestinal microbiota, and BSFL.

The gut microbiota influences host’s survival, development, immune, and metabolic functions through a plethora of secondary metabolites and molecules (17–20). However, in complex intestinal systems, determining which members of the microbiota influence the production of host secondary metabolites and the impact of these metabolites on the host remains a challenge. The germ-free animal model is commonly used to validate the role of the whole intestinal microbiota, whereas the monobacterial intestinal animal model is used to explore the function of a specific strain (21, 22). For instance, the gut microbial community of *Nasonia vitripennis* assisted in degrading the herbicide atrazine, which was verified using the germ-free *N. vitripennis* model (22). Another study confirmed the ciprofloxacin degradation level of five BSFL intestinal isolates by constructing germ-free and monobacterial intestinal BSFL models (23).

In our previous study, 10B was isolated from the BSFL gut, which was confirmed to possess a high extracellular enzymatic activity and improve the substrate conversion efficiency of BSFL (24). However, the specific mechanism of how the 10B regulates substrate conversion efficiency of BSFL remains unclear. Therefore, this study aims to (i) investigate how 10B influenced the bio-physiological phenotypes and substrate conversion efficiency of BSFL *via* gut microbiota modulation and (ii) explain the function of 10B on the BSFL through metabolic analysis of the germ-free and monobacterial intestinal BSFL. This study will better explain the functions of probiotics on the intestinal flora and its host metabolism, which provide theoretical support for probiotics’ practical applications in food waste conversion using BSFL.

## Materials and Methods

### Insect Husbandry and Determination of Bio-Physiological Indicators

Laboratory BSFL was provided by the culturing center of Henan Agricultural University. BSFL was reared until 3rd instar larvae at 30°C with 70% moisture content using wheat bran, and then continuously reared for 10 days using food waste mixed with ∼10% peanut shell powder (25). Then, the food waste conversion rate, food waste consumption rate, and survival rate of BSFL were determined as follows:


(1) SubstrateconversionrateofBSFL(%)=[(W-1W)2/W]1×100%

(2) SubstrateconsumptionrateofBSFL(%)=[(W-3W)4/W]3×100%

(3) SurvivalrateofBSFL(%)=(N/1N)2×100%

W_1_ and W_2_ are the dry weight of BSFL before and after rearing, respectively; W_3_ and W_4_ are the dry weight of the substrate before and after BSFL conversion, respectively. N_1_ and N_2_ are the BSFL survival number before and after rearing, respectively.

After 10 days of rearing, one part of BSFL was dried at 105°C in an oven for 48 h until the weight was stable for the following analysis. The dry weight of BSFL was measured with a thermogravimetric method (26). Then, they were grounded and passed through a 100-mesh sieve for nutrient determination. The crude fat and protein contents of BSFL were measured referencing the national standard of GB/T6433-20 and GB/T6432-1994, respectively (27). Another part of BSFL was dissected to collect their midgut tissues. Then, they were mixed with 1 ml of Tris-HC1 buffers (pH 7.5) and sterile glass beads. The mixture was shaken to grind the intestine thoroughly. After being centrifuged (8,000 r/min, 10 min), the supernatant was used to detect the activity of lipase, cellulase, and amylase, respectively, by the Lipase Activity Assay Kit (Solarbio, China), CL Activity Assay Kit (Solarbio, China), and AL Activity Assay Kit (Solarbio, China), according to manufacturer’s instruction (28). In addition, the protease enzyme activity of the midgut was determined using the Folin-phenol method (29).

### Microbial Community Analysis

Natural BSFLs were first reared on wheat bran until 3rd instar larvae as mentioned in the “Insect Husbandry and Determination of Bio-Physiological Indicators” section. Then, they were divided into two groups, one of which was reared with non-sterile food waste (CK), and the other was reared with non-sterile food waste and 10B (CK + 10B). After 10 days of incubation, BSFL gut and frass were collected separately in both groups, consisting of CK-gut, CK-frass, 10B-gut, and 10B-frass sets, which were subjected to extracting gDNA using the E.Z.N.A. Soil Kit (Omega Bio-Tek, United States) following the instructions. The primers 806R (5′-GGACTACHVGGGTWTCTAAT-3′) and 338F (5′-ACTCCTACGGGAGGCAGCAG-3′) were used to amplify the bacterial V3–V4 region of 16S rRNA genes. PCR reactions were performed as previously described (30). Purified PCR products were analyzed using the Illumina Miseq PE300 sequencing platform (Illumina, United States) (31). All sequencing data have been deposited in the NCBI Sequence Read Archive (SRA) under accession number PRJNA781802. The raw reads were processed according to the standard procedure of Majorbio Bio-Pharm Technology Co., Ltd. (Shanghai, China) (9).

### Germ-Free and Monobacterial Intestinal Black Soldier Fly Larvae Model Construction

In a 10-ml sterile centrifuge tube, 0.4 g of fresh BSFL eggs were weighed. To disperse the eggs, 1 ml of 2.7% NaClO solution was added by shaking for 1 min to aspirate them thoroughly. After that, 1 ml of Sporgon (Beijing Mingyangkehua Bio-Technology, China) was added. The mixture was shaken for 2 min to aspirate it completely. After leaving for 2 min, the BSFL eggs were washed two times with sterile water. Finally, they were placed on the ultraclean bench for 30 min for drying. Disinfected eggs were inoculated into the brain heart infusion (BHI) medium for further incubation for 24 h at 37°C. The culture was subsequently spread on the agar plates to verify the disinfection effect (32).

Subsequently, 30 sterile BSFLs were inoculated into 45 g autoclaved food wastes without or with 1 ml of 10B suspension (OD_600_ = 1.0) in which the germ-free and monobacterial intestinal BSFL were obtained, respectively. To verify the construction effect of the germ-free and monobacterial intestinal BSFL models, after 10 days of rearing at 37°C, germ-free and monobacterial intestinal BSFLs were dissected. On the one hand, 0.2 g of BSFL midgut and frass were suspended in 1 ml of sterile 0.85% NaCl, respectively, and 50 μl of suspension was spread on the BHI agar medium to verify the disinfection effect. On the other hand, 10 germ-free and monobacterial intestinal BSFLs were homogenized. The QIAamp Fast DNA Stool Mini Kit (QIAGEN, Germany) was used to extract the gDNA in both sets. The 16S rRNA gene was amplified using universal 1492R/27F primers (33).

### Metabolic Measurement and Data Analysis

To verify the effect of 10B on the host, 3rd instar BSFL were divided into three groups, namely, CK (natural BSFL fed with sterile food waste), GF (germ-free BSFL fed with sterile food waste), and GF + 10B (germ-free BSFL fed with sterile food waste and 10B). After 10 days of rearing, BSFLs in each group were used to determine bio-physiological parameters, such as survival rate, average dry weight of BSFL, substrate consumption rate, and substrate conversion rate as described in the “Insect Husbandry and Determination of Bio-Physiological Indicators” section. Furthermore, the metabolites of BSFL in CK, GF, and GF + 10B groups were all determined by a broadly targeted metabolism strategy. Wuhan MetWare Biotechnology Co., Ltd.^1^ assisted with the metabolite extraction, identification, detection, and quantification following the forementioned protocol (34). Each biological sample was checked in three replicates. All sample extracts were mixed and used as the quality control (QC). The metabolites were annotated by the Metware database (MWDB) and other publicly available databases (35).

Principal component analysis (PCA) was carried out to uncover the relationships among the samples based on the identified metabolites. The differentially accumulated metabolites (DAMs) between samples were distinguished by orthogonal partial least squares discriminant analysis (OPLS−DA) using the criteria of variable importance in the project (VIP) ≥ 1 and log_2_ (fold change) > 1. DAMs were posted to the corresponding metabolic pathways by the Kyoto Encyclopedia of Genes and Genomes (KEGG) database. KEGG enrichment analysis was then performed using a clusterProfiler (36). All the data analyses were performed in the R environment.^2^

### Vitamin Backfill Assays

Food waste (45 g) was autoclaved in glass bottles. By adding different vitamins or 10B alone, five experimental groups were set up, as follows: (i) GF: 30 germ-free BSFLs; (ii) GF + 10B: 30 germ-free BSFLs and 1 ml 10B (OD600 = 1.0); (iii) thiamine: 30 germ-free BSFLs and 1 ml thiamine (5 mg/ml); (iv) riboflavin: 30 germ-free BSFLs and 1 ml riboflavin (5 mg/ml); (v) biotin: 30 germ-free BSFLs and 1 ml biotin (5 mg/ml). The experiments were performed for 10 days in an incubator at 37°C. Then, BSFLs in each group were used to determine bio-physiological parameters, such as the survival rate, average dry weight of BSFL, substrate consumption rate, and substrate conversion rate, as described in the “Insect Husbandry and Determination of Bio-Physiological Indicators” section.

### Statistical Analysis

All experiments were performed with three replications. Data were exhibited as the mean ± SD. Tukey’s test was used to examine the results of bio-physiological indicators, colony counting, and microbial diversity indices. The significant correlation between the bio-physiological indicators and the relative abundance of dominant microbes was determined by Spearman’s analysis. SPSS 16.0 for Windows and the free online platform of Majorbio I-Sanger Cloud Platform^3^ were used to analyze the statistical differences. Spearman’s rank correlation analysis was used to determine the significant correlation among the relative abundance of dominant microbes.

## Results

### Effect of 10B on the Bio-Physiology of Black Soldier Fly Larvae

**FIGURE 1:**
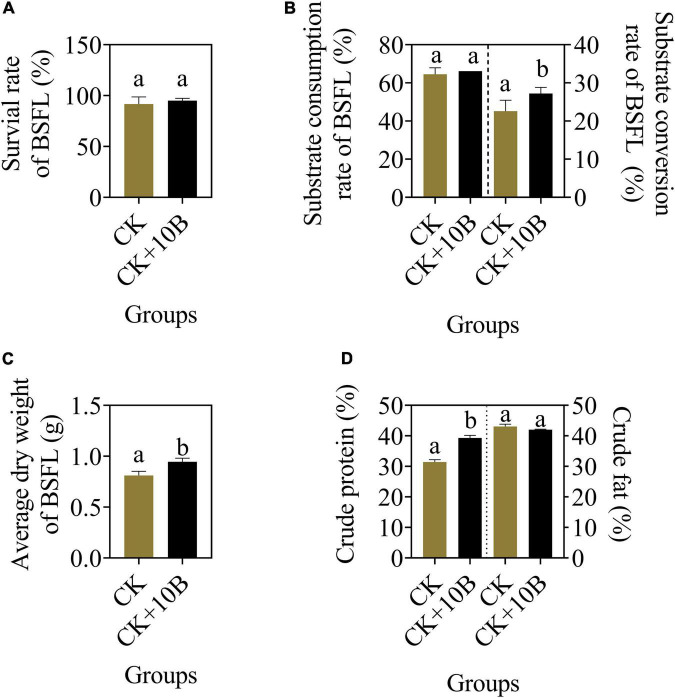
The bio-physiological parameters **(A)** survival rate, **(B)** substrate consumption/conversion rate, **(C)** average dry weight, and **(D)** crude protein/fat content of BSFL in the CK and CK + 10B groups. CK, natural BSFL fed on non-sterile food waste; CK + 10B, natural BSFL fed on non-sterile food waste and 10B. Data are presented as mean ± standard deviation (*n* = 3). Values with different letters mean significant differences at *p* < 0.05, as determined by Tukey’s test.

To evaluate the influence of 10B on the growth and substrate conversion efficiency of BSFL, they were inoculated into food waste. The larval survival rate and substrate consumption rate of BSFL were almost the same between the CK and CK + 10B groups (Figures 1A,B), indicating that the addition of 10B did not affect the survival of BSFL. In addition, the substrate conversion rate of BSFL in the CK + 10B group was improved by ∼5% compared to the CK group (Figure 1B), and thus the average dry weight of BSFL in the CK + 10B group was also increased (Figure 1C). The above results demonstrated that 10B greatly influences the substrate conversion process of BSFL. To further verify the influence of 10B on the nutritional value of BSFL, the nutrient composition of BSFL was presented. The crude protein content of BSFL in the CK + 10B group was increased ∼8% compared to the CK group, while there was no significant difference in crude fat content between them (Figure 1D). This result stated that 10B improved the protein synthesis process in BSFL. Moreover, the activities of the four intestinal digestive enzymes, namely, protease, amylase, cellulase, and lipase, increased by 65.17, 10.11, 37.62, and 31.61%, respectively in the CK + 10B group compared to the CK group (Supplementary Figure 1). This result further demonstrated that the addition of 10B enhanced the activity of the digestive enzymes, especially the protease activity, in the gut of BSFL, and thus elevated substance uptake and protein conversion ability of BSFL.

### Analysis of the Black Soldier Fly Larvae Gut and Frass Microbiota

**FIGURE 2:**
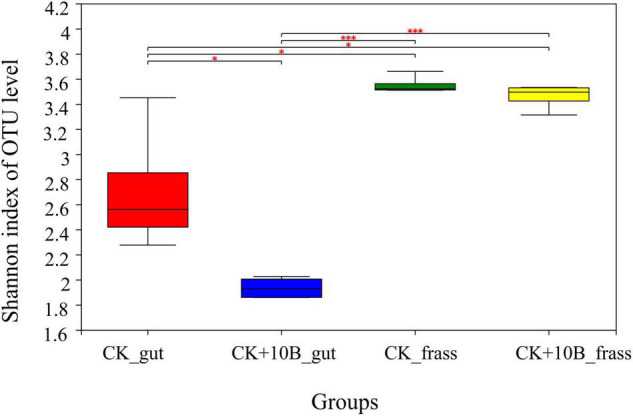
The Shannon index of gut microbiota in the CK_gut, CK+10B_gut, CK_frass, and CK+10B_frass groups. Pair-wise ANOVA was performed between different groups. “^∗^” indicates *p* < 0.05; “^∗∗∗^” indicates *p* < 0.001. CK_gut, intestinal microbiota of BSFL; CK_frass, frass microbiota of BSFL; 10B_gut, intestinal microbiota of BSFL inoculated with 10B; 10B_frass, frass microbiota of BSFL inoculated with 10B.

To explore the effect of 10B on the gut microbes of BSFL, the microbial composition of the gut and frass was characterized using Illumina Miseq sequencing after rearing for 10 days. After trimming and quality filtering, a total of 886,845 sequences with > 99.9% coverage for all samples were generated (Supplementary Figure 2A). The rarefaction curves further showed that all samples were almost approaching the saturation plateau, indicating that microbial communities were represented well (Supplementary Figure 2B). In general, the Shannon index value of frass was higher than that of the gut, indicating that microbial diversity of frass was higher than that of the gut. The Shannon index in both the CK_frass and CK + 10B_frass groups did not differ, while it was much lower in the CK + 10B_gut group compared to the CK_gut group (Figure 2). This result demonstrated that the addition of 10B altered the gut’s microbial diversity instead of the frass in BSFL.

**FIGURE 3:**
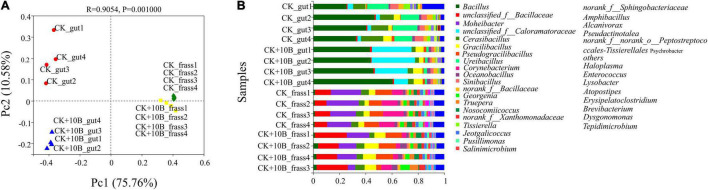
**(A)** Principal coordinate analysis (PCoA) score plot of different samples based on weighted UniFrac metrics. Three categories were clustered. **(B)** Gut microbial composition at the genus level (the relative abundance of microbes > 0.01) in different samples.

Dissimilarities of the above four groups were investigated using the principal coordinate analysis (PCoA). The first two axes totally explained 86.34% variance of species. In general, three categories were clustered, namely, (i) CK_frass and CK + 10B_frass, (ii) CK_gut, (iii) CK + 10B_gut (Figure 3A). The above results further demonstrated that the addition of 10B greatly influenced the gut microbial composition rather than the frass microbial composition of BSFL. The microbial composition analysis illustrated that the intestinal microbial composition differed from the frass at the genus level. In general, with the addition of 10B, the microbial composition has a massive change in the gut instead of frass. At the genus level, the relative abundance of *unclassified_of_Bacillaceae* and *Graclibacillus* in frass was significantly elevated, while the relative abundance of *Mohebacter* and *Corynebacterium* was markedly reduced. In addition, the relative abundance of *Bacillus*, *unclassified_of_Caloramatoraceae*, and *Gracilibacillus* was increased in the CK_gut group compared to the CK + 10B_gut group, whereas the relative abundance of *Cerasibacillus*, *Ureibacillus*, and *Sinibacillus* was decreased (Figure 3B). Notably, the relative abundance of *Bacillus* in the CK_gut group reached 48.39%, which was 12% higher than that in the 10B_gut group (Figure 3B), which indicated the colonization of 10B in the gut.

### Correlation Between Dominant Microbes at the Genus Level

Spearman’s correlation analysis showed that *Bacillus* displayed positive correlation coefficients with *unclassified_of_Caloramatoraceae* and *Gracilibacillus*, whereas it presented negative correlations with *Cerasibacillus*, *Ureibacillus*, and *Sinibacillus* (*p* < 0.05). This result indicated that 10B might also affect host metabolism by influencing the relative abundance of these mentioned microbes (Table 1).

**TABLE 1:** Spearman’s correlation coefficients among the relative abundance of the dominant microbes.

	*Bacillus*	*Unclassified_f_Caloramatoraceae*	*Gracilibacillus*	*Cerasibacillus*	*Ureibacillus*	*Sinibacillus*
*Bacillus*	1					
*Unclassified_f_Caloramatoraceae*	0.4286	1				
*Gracilibacillus*	0.6667	0.1429	1			
*Cerasibacillus*	−0.4286	−0.9524	−0.1667	1		
*Ureibacillus*	−0.5000	−0.6905	−0.5714	0.7857	1	
*Sinibacillus*	−0.7143	−0.7619	−0.3095	0.8095	0.7381	1

*All the correlation is significant at the p < 0.05 level.*

### Correlation Between the Microbial Communities and Bio-Physiological Variables

**FIGURE 4:**
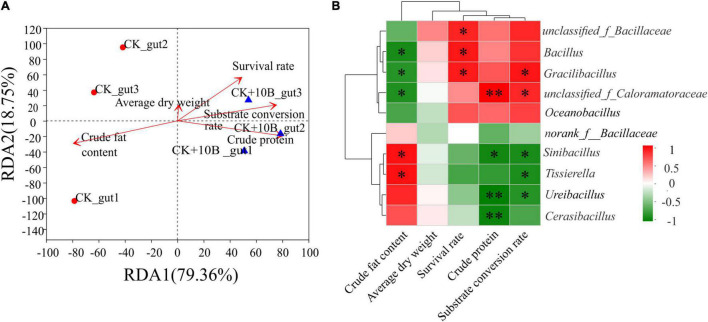
**(A)** Redundancy analysis (RDA) of microbial community structure and physiological indexes of BSFL. Arrows indicate the magnitude and direction of measurable variables correlated to microbial community structure. **(B)** Pearson’s correlation between the relative abundance of physiological indexes and the 10 most dominant microbes in gut microbes of the CK and CK + 10B groups. CK, natural BSFL fed on non-sterile food waste; CK + 10B, natural BSFL fed on non-sterile food waste and 10B. The symbol ^∗∗^ means the correlation is significant at the *p* < 0.01 level. The symbol ^∗^ means the correlation is significant at the *p* < 0.05 level.

The relationship between the microbial community structure and physiological indexes, including the survival rate, substrate conversion rate, dry weight, crude fat content, and crude protein content of BSFL, was revealed by redundancy analysis (RDA). As shown in Figure 4A, the survival rate, substrate conversion rate, dry weight, and crude protein content were significantly and positively correlated to the bacterial community structure of the 10B + CK_gut group. In contrast, crude fat content presented a significantly negative correlation with the microbial community structure of the CK_gut group (Figure 4A). This result demonstrated that 10B influenced the metabolism of BSFL by affecting the structure of the microbial community. To further understand the specific genera’s contribution to the BSFL’s metabolism, the relationship between the 10 most dominant microbes and physiological indexes was analyzed using Pearson’s correlation analysis. Notably, there was a high positive correlation between *unclassified_of_Caloramatoraceae*, *Bacillus*, and *Gracillibacillus* and the survival rate of BSFL, with correlation coefficients of 0.825, 0.872, 0.861, respectively (*p* ≤ 0.05). In addition, *Gracillibacillus* also had significant correlations with the substrate conversion rate of BSFL (correlation coefficients = 0.883, *p* ≤ 0.05). *Unclassified_of_Caloramatoraceae* showed significant positive correlations with the crude protein content and substrate conversion rate, and the correlation coefficients were up to 0.963 (*p* ≤ 0.01) and 0.814 (*p* ≤ 0.05), respectively (Figure 4B). The above findings suggested that 10B directly impacted the host’s survival and influenced BSFL’s metabolism by altering the abundance of other intestinal microbes.

### Influence of 10B on the Growth Performance of Monobacterial Intestinal Black Soldier Fly Larvae

**FIGURE 5:**
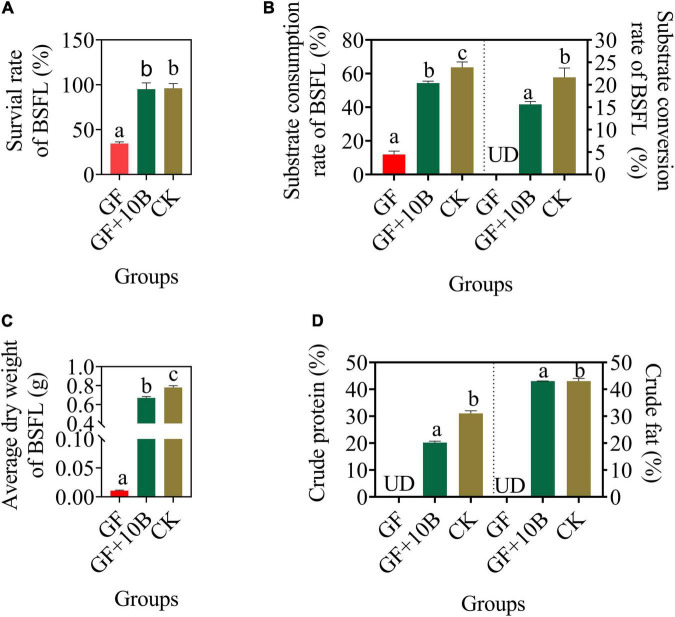
The bio-physiological parameters, namely, **(A)** survival rate, **(B)** substrate consumption/conversion rate, **(C)** average dry weight, and **(D)** crude protein/fat content of BSFL in the GF, GF + 10B, CK groups. GF, germ-free BSFL fed on sterile food waste; GF + 10B, germ-free BSFL fed on sterile food and 10B; CK, natural BSFL fed on sterile food waste. Data are shown as mean ± standard deviation (*n* = 3). Values with different letters mean significant differences at *p* < 0.05, as determined by Tukey’s test. UD, undetected.

To further evaluate the impact of 10B on BSFL growth and substrate conversion efficiency, it was inoculated into the sterile BSFL system to construct a monobacterial intestinal BSFL model. The germ-free BSFL failed to develop normally, with a survival rate of 34%, whereas the larval survival rate of BSFL reared with 10B only recovered to 100% after 10 days of rearing (Figure 5A). Besides, the substrate consumption rate of BSFL in the GF + 10B and CK groups was increased to 54.35% and 63.67%, respectively, while it only reached 11.91% in the GF group (Figure 5B). The substrate conversion rate of BSFL in the GF + 10B group was elevated to 15.64%, which was only ∼5% lower than BSFL in the CK group (Figure 5B). As a result, the average dry weight of BSFL was increased from 0.01 g (GF) to 0.67 g (GF + 10B) (Figure 5C). In addition, the crude protein and crude fat contents of BSFL in the GF + 10B group reached 20.19% and 43.02%, respectively, while they could not be detected in germ-free BSFL (Figure 5D). Moreover, the activities of four intestinal digestive enzymes, namely, protease, amylase, cellulase, and lipase, were significantly higher in the GF + 10B group than those in the GF group (*p* < 0.05), but lower than those in the CK group (Supplementary Figure 4). Based on the above results, we hypothesized that 10B could provide BSFL with the nutrients required for survival and facilitate the substrate synthesis and conversion process of BSFL.

### Metabolic Analysis of Black Soldier Fly Larvae Under Different Rearing Conditions

**FIGURE 6:**
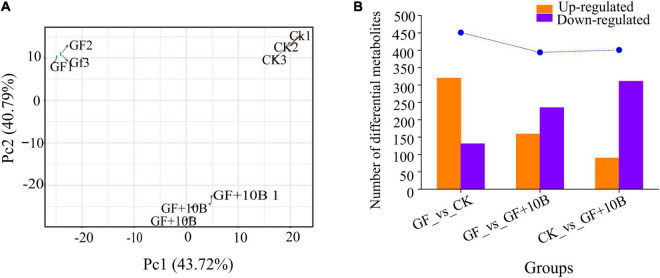
**(A)** Principal component analysis of the detected metabolites in the BSFL with three biological replicates. **(B)** Number of differentially accumulated metabolites (DAMs) of BSFL under different rearing conditions. GF, germ-free BSFL fed on sterile food waste; GF + 10B, germ-free BSFL fed on sterile food and 10B; CK, natural BSFL fed on sterile food waste.

To further explore the effect of 10B on BSFL, systematic metabolic profiling of BSFLs in the GF, GF + 10B, and CK groups was carried out. A total of 917 metabolites were identified in the three groups (Supplementary Sheet 1). PCA analysis of the detected metabolites showed that three biological replicates of each sample tended to group together, indicating that the generated metabolic data were highly reproducible. Meanwhile, in a two-dimensional plot, the dispersion pattern of the three groups showed significant differences among metabolite profiles (Figure 6A). To investigate the metabolic differences of BSFL under different rearing conditions, pair-wise comparisons of the metabolites were used to identify the DAMs (Figure 6B), the details of which were listed in Supplementary Sheets 2–4. A similar number of DAMs were identified when comparing the GF_vs._GF + 10B (394) and CK_vs._GF + 10B groups (401). A total of 451 DAMs were discovered in the comparison of the GF_vs._CK group. There are more upregulated DAMS than the downregulated DAMs in the comparison of GF vs. CK groups. In contrast, the number of downregulated DAMs was greater than that of upregulated in the comparison of the GF_vs._GF + 10B and CK_vs._GF + 10B groups. The DAMs identified in the pair−wise comparisons accounted for 42.97–49.18% of all detected metabolites, verifying the rich diversity of metabolites exhibited in BSFL rearing under the three conditions described above. In addition, the highest number of DAMs was detected in the comparison of the GF_vs._CK groups (Figure 6B), indicating a significant influence of intestinal microbes on BSFL.

### Key Kyoto Encyclopedia of Genes and Genomes Pathways in Shaping the Differential Metabolites

**FIGURE 7:**
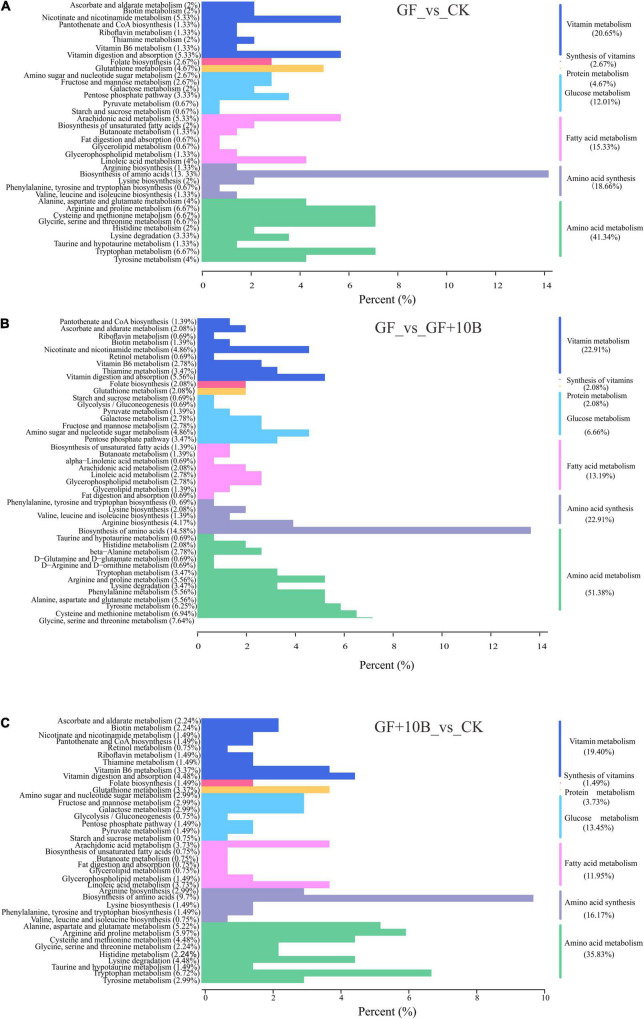
The KEGG enrichment analysis of the DAMs between **(A)** GF and CK. **(B)** GF and GF + 10B. **(C)** CK and GF + 10B. GF, germ-free BSFL fed on sterile food waste; GF + 10B, germ-free BSFL fed on sterile food and 10B; CK, natural BSFL fed on sterile food waste.

The KEGG classification analysis revealed that a large proportion of identified DAMs was involved in critical biological pathways, such as vitamin metabolism, vitamin synthesis, protein metabolism, glucose metabolism, fatty acid metabolism, amino acid synthesis, and amino acid metabolism (Figure 7). Notably, the GF_vs._CK group had 23.32% DAMs in the vitamin synthesis and metabolism classification (Figure 7A), indicating vitamin synthetic and metabolic differences between the GF and CK groups. Although 24.99% DAMs in the GF_vs._GF + 10B group were related to the synthesis and metabolism of vitamins (Figure 7B), 10B influenced vitamin synthesis and metabolism in BSFL. Moreover, the CK_vs._GF + 10B group was enriched in fewer DAMs (20.89%) than the other two groups (Figure 7C), implying that 10B plays an important role in BSFL *via* vitamins. Besides, DAMs were enriched in 60% of amino acid synthesis metabolism classification in the GF_vs._CK group (Figure 7A), indicating that gut microbes strongly affected the amino acid synthesis and metabolism of BSFL. In addition, 74.29% of DAMs were clustered with the amino acid synthesis and metabolism in the GF_vs._GF + 10B group (Figure 7B), suggesting that 10B had a more significant influence on BSFL amino acid synthesis and metabolism in the GF_vs._GF + 10B group. Furthermore, 52% of DAMs were found to be related to the amino acid synthetic and metabolic process in comparison to the CK_vs._GF + 10B group (Figure 7C), indicating that 10B mitigates to some extent the differences in the amino acid synthesis and metabolism of the host. These results also demonstrated that 10B played a vital role in BSFL by affecting the amino acid synthesis and metabolism process. Meanwhile, there were differences in the classification of glucose metabolism, fatty acid metabolism, and protein metabolism among these three groups (Figure 7). This result also implied that 10B might also play a role in BSFL by affecting the above metabolic processes. Furthermore, a total of 12 classes of differentially accumulated vitamins were identified, of which biotin, riboflavin, and thiamine were downregulated in the GF_vs._GF + 10B and GF_vs._CK groups and upregulated in the CK_vs._GF + 10B group (Table 2). This result suggested that 10B may increase the survival of BSFL by altering the uptake and metabolism of the three vitamins mentioned above.

**TABLE 2:** Functional analyses of identified differentially accumulated metabolites related to vitamins.

		Log_2_ (Fold changes)		
Number of DAMs	Name of DAMs	GF_vs._CK	GF_vs._GF + 10B	CK_vs._GF + 10B
MEDN0132	Biotin	−4.99 ± 0.16	−1.23 ± 0.30	3.76 ± 0.20
MEDN0244	Orotic acid	1.74 ± 0.60	−	−2.00 ± 0.63
MEDN0245	Pantothenate	−4.16 ± 0.05	−5.42 ± 0.04	−1.25 ± 0.04
MEDP0240	Pyridoxine	−	−	−1.55 ± 0.26
MEDN0248	Nicotinuric acid	−3.07 ± 0.13	−2.78 ± 0.41	−
MEDP0242	Nicotinamide	−3.88 ± 0.15	−3.24 ± 0.14	−
MEDP0244	All-trans-13,14-dihydroretinol	−	−4.34 ± 0.33	−3.25 ± 0.20
MEDP0246	Nicotinic Acid	−1.35 ± 0.11	−	1.17 ± 0.05
MEDP0248	N-Methylnicotinamide	−	−13.24 ± 0.22	−13.24 ± 0.22
MEDP0250	Riboflavin	−4.02 ± 0.03	−2.30 ± 0.10	1.72 ± 0.16
MEDP0514	Thiamine	−6.14 ± 0.46	−4.31 ± 0.52	1.84 ± 0.23
MEDP1876	Methyl nicotinic acid	−2.74 ± 0.17	−2.98 ± 0.24	−

*All data are presented as mean standard deviation (n = 3).*

### Role of 10B in Black Soldier Fly Larvae Substrate Conversion Process

To further verify our hypothesis, 10B, thiamine, riboflavin, and biotin were added to the food waste, respectively. With the addition of 10B or riboflavin alone, the survival rate of BSFL reached 100% and 98%, respectively, which amounted to that of the CK group. This result confirmed that 10B improved the BSFL survival rate through the provision of riboflavin to the host. Although the substrate consumption rate of BSFL reached 50% after the supplement of riboflavin, which was only 10% lower than that of the CK and GF + 10B groups, the substrate conversion rate was much lower than that of BSFL in the CK and GF + 10B groups, resulting in a much lower dry weight of BSFL in the riboflavin-added group than in the CK and GF + 10B groups. This result indicates that 10B also plays a critical role in converting substrate to nutrients.

## Discussion

The BSFL can convert organic wastes into insect biomass as an alternative protein source (14–16). In this study, we illustrated the co-conversion performance of BSFL on food waste with and without probiotic 10B, as well as the influence of 10B addition on BSFL microbial composition and metabolic process. By analyzing bio-physiological parameters and gut microbiota together, the interaction between the gut microbiome and the food waste conversion process was explained. In addition, by analyzing the bio-physiological characterization and metabolites of germ-free BSFL, monobacterial intestinal BSFL, and natural BSFL, we not only identified that 10B determined the survival of 10B *via* providing riboflavin to the host but also illustrated that 10B increased the protein content by influencing the synthetic and metabolic processes of amino acids in BSFL. Taken together, our results provide a theoretical reference for the mechanisms by which probiotics promote the conversion of more substrates into insect proteins.

In this study, process performance can be further improved by inoculating 10B to BSFL, thus increasing dry larval biomass and food waste conversion by 13.8% and 17.08%, respectively (Figures 1B,C), without influencing the substrate consumption rate (Figure 1C). Hence, 10B can provide economic benefits to food production and animal feeds for BSFL. The results of our research are lower than those reported for chicken manure inoculated with *B. subtilis* and BSFL, which showed a 12.7% increase in bioconversion and a 13.4% increase in waste reduction rate compared to BSFL without bacteria inoculation group (15). A similar study also showed that with the addition of *B. subtilis*, BSFL showed a 22% increase in biomass (14). The addition of 10B to BSFL did not significantly affect the fat content of BSFL, but the protein content increased dramatically by ∼8% (Figure 1D). This result stays in line with a previous study that showed *L. buchneri* increased the protein content of BSFL by 50.4–55.3%, without a noticeable influence on the fat content of BSFL (16).

Although previous studies have explored the influence of functional bacteria on BSFL bio-physiological parameters, their impact on the gut microbiota has never been explored. In this study, the provision of 10B to BSFL resulted in a significant change in the diversity and composition of gut microbiota instead of frass microbiota (Figures 2, 3). This result indicated that 10B mainly colonized the gut and changed the gut microbiota, thus improving substrate conversion efficiency and protein conversion process. Interestingly, Pearson’s correlation analysis further confirmed that *Gracilibacillus* was significantly and positively correlated with substrate conversion rate, while *unclassiffied_of_Caloramatoraceae* was positively correlated with substrate conversion rate and crude protein (Figure 4B). *Gracilibacillus* was reported to hydrolyze macromolecular gelatin and proteins highly efficiently (37), which is also consistent with the findings in this study that the protease and amylase activity was improved by 65.17% and 10.11%, respectively, after the addition of 10B (Supplementary Figure 1). Besides, this is the first report of *Caloramatoracea* associated with substrate conversion to the best of our knowledge.

**FIGURE 8:**
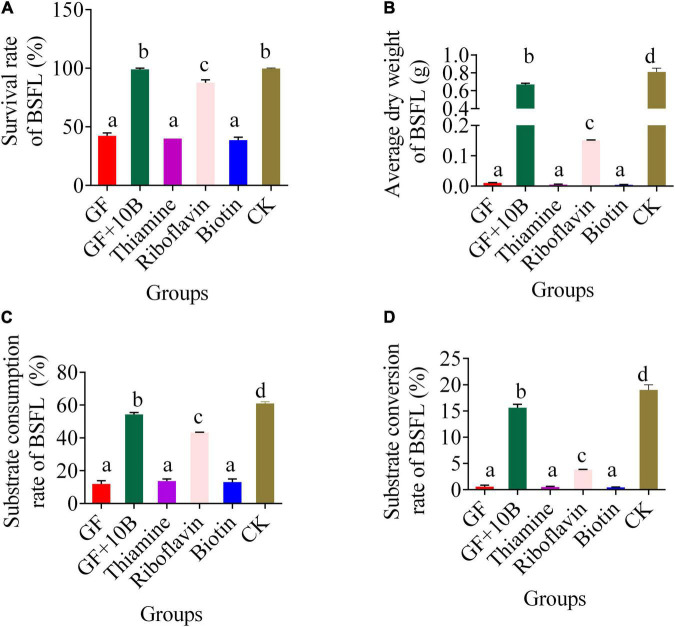
The bio-physiological parameters, namely, **(A)** survival rate, **(B)** average dry weight, **(C)** substrate consumption rate, and **(D)** substrate conversion rate of BSFL in the GF, GF + 10B, thiamine, riboflavin, biotin, and CK groups. GF, germ-free BSFL fed on sterile food waste; GF + 10B, germ-free BSFL fed on sterile food and 10B; thiamine, germ-free BSFL fed on sterile food waste and 1 ml thiamine (5 mg/ml); riboflavin, germ-free BSFL fed on sterile food waste and 1 ml riboflavin (5 mg/ml); biotin, germ-free BSFL fed on sterile food waste and 1 ml biotin (5 mg/ml); CK, natural BSFL fed on sterile food waste. Data are presented as mean ± standard deviation (*n* = 3). Values with different letters mean significant differences at *p* < 0.05, as determined by Tukey’s test.

To verify 10B’s specific function on the host, germ-free BSFL and monobacterial intestinal BSFL models were constructed. The addition of 10B restored the survival rate of BSFL to 87.7% (Figure 8A). The KEGG classification analysis of DAMs indicated that 10B might affect BSFL through vitamin metabolism (Figure 7). This result stays in line with previous studies that gut microbial communities often deliver metabolic benefits to hosts through the production of vitamins, thus determining their health (38). Furthermore, biotin, riboflavin, and thiamine were downregulated in the GF_vs._GF + 10B and GF_vs._CK groups and upregulated in the CK_vs._GF + 10B group (Table 2). This result is also consistent with previous studies that gut microbiota supplies its host with B vitamins, such as biotin, folate, pantothenic acid, pyridoxine, riboflavin, and thiamine (39–42). In addition, the vitamin backfill assay verified that 10B provides riboflavin to the host, thereby enabling the survival and growth of BSFL (Figure 8). Numerous studies have confirmed that several intestinal bacterial genera can synthesize B vitamins. For instance, *Bacteroides* is associated with the production of riboflavin, niacin, pantothenate, and pyridoxine. *Clostridium* is associated with the synthesis of folate, cobalamin, niacin, and thiamine (43), while *Bifidobacterium* is associated with folate synthesis (44).

Although monobacterial intestinal BSFL can be used to validate the function of one specific gut probiotic to some extent, the gut microbiota is a homeostatic and collaborative environment. The addition of functional bacteria may alter the structure of their gut microbial community structure by increasing the proportion of other functional bacteria rather than acting directly on their own. Therefore, further studies will focus on analyzing DAMs of BSFL gut microbes and their effects on the host.

## Conclusion

In this study, the comprehensive effect of probiotic 10B on the physiology, intestinal microbial community, and metabolism of BSFL was investigated. The larval biomass weight, bioconversion rate, and protein content of BSFL were improved to 0.95 g, 27.22% and 39.3%, respectively, when it was fed with food wastes and 10B. Illumina Miseq analysis showed that 10B increased substrate conversion efficiency by modulating the relative abundance of *Bacillus*, *unclassified_of_Caloramatoraceae*, and *Gracilibacillus* in the BSFL gut. Metabolic analysis on germ-free, monobacterial intestinal, and natural BSFL revealed that 10B determined the survival of BSFL through the provision of riboflavin and regulated the protein/amino acid conversion process of BSFL, thus increasing the BSFL protein content. This study provides a theoretical reference and a new strategy for using probiotic bacteria to improve substrate conversion efficiency and insect protein content. In future studies, we will focus on developing more efficient conversion probiotics and evaluating their practical application in the conversion of different organic wastes using BSFL.

## Data Availability Statement

The datasets presented in this study can be found in online repositories. The names of the repository/repositories and accession number(s) can be found in the article/Supplementary Material.

## Author Contributions

YP did most of the experiments, analyzed the data, and contributed to writing and revising the manuscript. SZ extracted DNA of gut microbiota and analyzed the Illumina Miseq sequencing data. XC and JZ helped with insect husbandry and determination of bio-physiological indicators of BSFL. HN and MS contributed to revising the manuscript. HL and XL helped with the germ-free and monobacterial intestinal BSFL models construction. HC contributed to the metabolic data analysis. SY provided overall directions and assisted in revising the manuscript. All authors approved submission of this manuscript to Frontiers in Nutrition.

## Conflict of Interest

The authors declare that the research was conducted in the absence of any commercial or financial relationships that could be construed as a potential conflict of interest.

## Publisher’s Note

All claims expressed in this article are solely those of the authors and do not necessarily represent those of their affiliated organizations, or those of the publisher, the editors and the reviewers. Any product that may be evaluated in this article, or claim that may be made by its manufacturer, is not guaranteed or endorsed by the publisher.

## Footnotes

1
https://www.metware.cn/

2
https://www.r-project.org/

3
https://cloud.majorbio.com/

## References

[1] Cawthorn D-M Hoffman LC. The role of traditional and non-traditional meat animals in feeding a growing and evolving world. *Anim Front.* (2014) 4:6–12. 10.2527/af.2014-0027 32704858

[2] Tanga CM Waweru JW Tola YH Onyoni AA Khamis FM Ekesi S Organic waste substrates induce important shifts in gut microbiota of black soldier fly (*Hermetia illucens* L.): coexistence of conserved, variable, and potential pathogenic microbes. *Front Microbiol.* (2021) 12:635881. 10.3389/fmicb.2021.635881 33643270 PMC7907179

[3] Matassa S Boon N Pikaar I Verstraete W. Microbial protein: future sustainable food supply route with low environmental footprint. *Microb Biotechnol.* (2016) 9:568–75. 10.1111/1751-7915.12369 27389856 PMC4993174

[4] Selaledi L Mbajiorgu CA Mabelebele M. The use of yellow mealworm (*T. molitor*) as alternative source of protein in poultry diets: a review. *Trop Anim Health Prod.* (2020) 52:7–16. 10.1007/s11250-019-02033-7 31392553

[5] Kim T-K Yong HI Kim Y-B Kim H-W Choi Y-S. Edible insects as a protein source: a review of public perception, processing technology, and research trends. *Food Sci Anim Resour.* (2019) 39:521–40. 10.5851/kosfa.2019.e53 31508584 PMC6728817

[6] Gasco L Acuti G Bani P Dalle Zotte A Danieli PP De Angelis A Insect and fish by-products as sustainable alternatives to conventional animal proteins in animal nutrition. *Ital J Anim Sci.* (2020) 19:360–72.

[7] De Smet J Wynants E Cos P Van Campenhout L. Microbial community dynamics during rearing of black soldier fly larvae (*Hermetia illucens*) and impact on exploitation potential. *Appl Environ Microbiol.* (2018) 84:AEM.02722-17. 10.1128/AEM.02722-17 29475866 PMC5930328

[8] Lalander C Diener S Zurbrügg C Vinnerås B. Effects of feedstock on larval development and process efficiency in waste treatment with black soldier fly (*Hermetia illucens*). *J Clean Prod.* (2019) 208:211–9. 10.1016/j.jclepro.2018.10.017

[9] Ao Y Yang C Wang S Hu Q Yi L Zhang J Characteristics and nutrient function of intestinal bacterial communities in black soldier fly (*Hermetia illucens* L.) larvae in livestock manure conversion. *Microb Biotechnol.* (2020) 14:886–96. 10.1111/1751-7915.13595 32449587 PMC8085981

[10] Zhan S Fang G Cai M Kou Z Xu J Cao Y Genomic landscape and genetic manipulation of the black soldier fly *Hermetia illucens*, a natural waste recycler. *Cell Res.* (2020) 30:50–60. 10.1038/s41422-019-0252-6 31767972 PMC6951338

[11] Jiang C-L Jin W-Z Tao X-H Zhang Q Zhu J Feng S-Y Black soldier fly larvae (*Hermetia illucens*) strengthen the metabolic function of food waste biodegradation by gut microbiome. *Microb Biotechnol.* (2019) 12:528–43. 10.1111/1751-7915.13393 30884189 PMC6465238

[12] Feng P Ye Z Han H Ling Z Li X. Tibet plateau probiotic mitigates chromate toxicity in mice by alleviating oxidative stress in gut microbiota. *Commun Biol.* (2020) 3:242. 10.1038/s42003-020-0968-3 32415160 PMC7229148

[13] Kyritsis GA Augustinos AA Cáceres C Bourtzis K. Medfly gut microbiota and enhancement of the sterile insect technique: similarities and differences of *Klebsiella oxytoca* and *Enterobacter* sp. AA26 probiotics during the larval and adult stages of the VIENNA 8D53+ genetic sexing strain. *Front Microbiol.* (2017) 8:2064. 10.3389/fmicb.2017.02064 29163379 PMC5663728

[14] Yu G Cheng P Chen Y Li Y Yang Z Chen Y inoculating poultry manure with companion bacteria influences growth and development of black soldier fly (Diptera: Stratiomyidae) larvae. *Environ Entomol.* (2011) 40:30–5. 10.1603/EN10126 22182608

[15] Xiao X Mazza L Yu Y Cai M Zheng L Tomberlin JK Efficient co-conversion process of chicken manure into protein feed and organic fertilizer by *Hermetia illucens* L. (Diptera: Stratiomyidae) larvae and functional bacteria. *J Environ Manag.* (2018) 217:668–76. 10.1016/j.jenvman.2018.03.122 29654970

[16] Somroo AA Ur Rehman K Zheng L Cai M Xiao X Hu S Influence of *Lactobacillus buchneri* on soybean curd residue co-conversion by black soldier fly larvae (*Hermetia illucens*) for food and feedstock production. *Waste Manag.* (2019) 86:114–22. 10.1016/j.wasman.2019.01.022 30902235

[17] El Sheikha AF. Tracing insect pests: is there new potential in molecular techniques? *Insect Mol Biol.* (2019) 28:759–72. 10.1111/imb.12601 31125162

[18] El Sheikha AF Menozzi P. Potential geo-tracing tool for migrant insects by using 16S rDNA fingerprinting of bacterial communities by PCR-DGGE. *Int J Trop Insect Sci.* (2019) 39:9–16. 10.1007/s42690-019-00002-z

[19] Jacoby RP Koprivova A Kopriva S. Pinpointing secondary metabolites that shape the composition and function of the plant microbiome. *J Exp Bot.* (2020) 72:57–69. 10.1093/jxb/eraa424 32995888 PMC7816845

[20] Taghinasab M Jabaji S. Cannabis microbiome and the role of endophytes in modulating the production of secondary metabolites: an overview. *Microorganisms.* (2020) 8:355. 10.3390/microorganisms8030355 32131457 PMC7143057

[21] Shropshire JD Van Opstal EJ Bordenstein SR. An optimized approach to germ-free rearing in the jewel wasp Nasonia. *PeerJ.* (2016) 4:e2316. 10.7717/peerj.2316 27602283 PMC4991892

[22] Wang G-H Berdy BM Velasquez O Jovanovic N Alkhalifa S Minbiole KPC Changes in microbiome confer multigenerational host resistance after sub-toxic pesticide exposure. *Cell Host Microbe.* (2020) 27:213–24.e17. 10.1016/j.chom.2020.01.009 32023487

[23] Yang C Ma S Li F Zheng L Tomberlin JK Yu Z Characteristics and mechanisms of ciprofloxacin degradation by black soldier fly larvae combined with associated intestinal microorganisms. *Sci Total Environ.* (2021) 811:151371. 10.1016/j.scitotenv.2021.151371 34740641

[24] Xiang C Sijie Z Tingting L Jiran Z Hongge C Sen Y. Identification and enzyme profiling of black soldier fly egg commensal *Bacillus velezensis* and its effect on food waste bioconversion. *Acta Microbiol Sin.* (2021) 61:2121–35.

[25] Cheng JYK Chiu SLH Lo IMC. Effects of moisture content of food waste on residue separation, larval growth and larval survival in black soldier fly bioconversion. *Waste Manag.* (2017) 67:315–23. 10.1016/j.wasman.2017.05.046 28587803

[26] Nasser RA Salem MZM Hiziroglu S Al-Mefarrej HA Mohareb AS Alam M Chemical analysis of different parts of date palm (*Phoenix dactylifera* L.) using ultimate, proximate and thermo-gravimetric techniques for energy production. *Energies.* (2016) 9:374. 10.3390/en9050374

[27] Yu T Chen Y-K Chen X-M Abdallah G Guo Z-X Zhao Y-L The effect of oxidized fish oil on lipid metabolism in *Rhynchocypris lagowski* Dybowski. *Aquac Rep.* (2020) 17:100388. 10.1016/j.aqrep.2020.100388

[28] Gao Y Hou L Wang Y Guo S Yuan D Jiang YN Octreotide alleviates pancreatic damage caused by paraquat in rats by reducing inflammatory responses and oxidative stress. *Environ Toxicol Pharmacol.* (2020) 80:103456. 10.1016/j.etap.2020.103456 32673753

[29] Yun-Feng H Ren-Qiang L Ai-Jun S Shan X Yun Z Zhen-Hua Z. Properties of extracellular protease of microbe DH-2 from mangrove and optimization of enzyme producing conditions. *Biotechnol Bull.* (2018) 34:120–7.

[30] Zhang XX Zhang JZ Jiang LL Yu X Zhu HW Zhang JL Black soldier fly (*Hermetia illucens*) larvae significantly change the microbial community in chicken manure. *Curr Microbiol.* (2021) 78:303–15. 10.1007/s00284-020-02276-w 33141316

[31] Chen K Huang G Li Y Zhang X Lei Y Li Y Illumina miSeq sequencing reveals correlations among fruit ingredients, environmental factors, and AMF Communities in three *Lycium barbarum* producing regions of China. *Microbiol Spectr.* (2022) 10:e0229321. 10.1128/spectrum.02293-21 35234495 PMC8941938

[32] Mei H Li C Li X Hu B Lu L Tomberlin JK Characteristics of tylosin and enrofloxacin degradation in swine manure digested by black soldier fly (*Hermetia illucens* L.) larvae. *Environ Pollut.* (2022) 293:118495. 10.1016/j.envpol.2021.118495 34785289

[33] Zhang H Chu W Sun J Liu Z Huang W-C Xue C Combining cell surface display and DNA-shuffling technology for directed evolution of streptomyces phospholipase D and synthesis of phosphatidylserine. *J Agric Food Chem.* (2019) 67:13119–26. 10.1021/acs.jafc.9b05394 31686506

[34] Huang H-Y Ren Q-Q Lai Y-H Peng M-Y Zhang J Yang L-T Metabolomics combined with physiology and transcriptomics reveals how *Citrus grandis* leaves cope with copper-toxicity. *Ecotoxicol Environ Saf.* (2021) 223:112579. 10.1016/j.ecoenv.2021.112579 34352583

[35] Cao H Ji Y Li S Lu L Tian M Yang W Extensive metabolic profiles of leaves and stems from the medicinal plant *Dendrobium officinale* Kimura et Migo. *Metabolites.* (2019) 9:215. 10.3390/metabo9100215 31590300 PMC6835975

[36] Zheng J Zhang T Guo W Zhou C Cui X Gao L Integrative analysis of multi-omics identified the prognostic biomarkers in acute myelogenous leukemia. *Front Oncol.* (2020) 10:591937. 10.3389/fonc.2020.591937 33363022 PMC7758482

[37] Ma H Beadham I Ruan W Zhang C Deng Y. Enhancing rice straw compost with an amino acid-derived ionic liquid as additive. *Bioresour Technol.* (2022) 345:126387. 10.1016/j.biortech.2021.126387 34838960

[38] Engel P Moran NA. The gut microbiota of insects – diversity in structure and function. *FEMS Microbiol Rev.* (2013) 37:699–735. 10.1111/1574-6976.12025 23692388

[39] Piper MDW Blanc E Leitão-Gonçalves R Yang M He X Linford NJ A holidic medium for *Drosophila melanogaster*. *Nat Methods.* (2014) 11:100–5. 24240321 10.1038/nmeth.2731 PMC3877687

[40] Wong AC-N Dobson AJ Douglas AE. Gut microbiota dictates the metabolic response of *Drosophila* to diet. *J Exp Biol.* (2014) 217:1894–901. 10.1242/jeb.101725 24577449 PMC4037322

[41] Sannino DR Dobson AJ Edwards K Angert ER Buchon N. The *Drosophila melanogaster* gut microbiota provisions thiamine to its host. *mBio.* (2018) 9:e00155-18. 10.1128/mBio.00155-18 29511074 PMC5845000

[42] Alrubaye HS Kohl KD Ishaq SL. Abundance and compositions of B-vitamin-producing microbes in the mammalian gut vary based on feeding strategies. *mSystems.* (2021) 6:e313–21. 10.1128/mSystems.00313-21 34463576 PMC12338137

[43] Magnúsdóttir S Ravcheev D De Crécy-Lagard V Thiele I. Systematic genome assessment of B-vitamin biosynthesis suggests co-operation among gut microbes. *Front Genet.* (2015) 6:148. 10.3389/fgene.2015.00148 25941533 PMC4403557

[44] D’Aimmo MR Mattarelli P Biavati B Carlsson NG Andlid T. The potential of bifidobacteria as a source of natural folate. *J Appl Microbiol.* (2012) 112:975–84. 10.1111/j.1365-2672.2012.05261.x 22335359

