# Supplementary material for: Bacillus velezensis EEAM 10B Strengthens Nutrient Metabolic Process in Black Soldier Fly Larvae (Hermetia illucens) via Changing Gut Microbiome and Metabolic Pathways
Source: Front Nutr. 2022 May 19;9:880488. doi: 10.3389/fnut.2022.880488 (PMC9161358; doi:10.3389/fnut.2022.880488)
Supplement: Supplementary file 5 [file Image_1.pdf]

Supplementary Images for

***Bacillus velezensis* EEAM 10B strengthens nutrient metabolic process in black soldier fly larvae (*Hermetia illucens*) via changing gut microbiome and metabolic pathways**

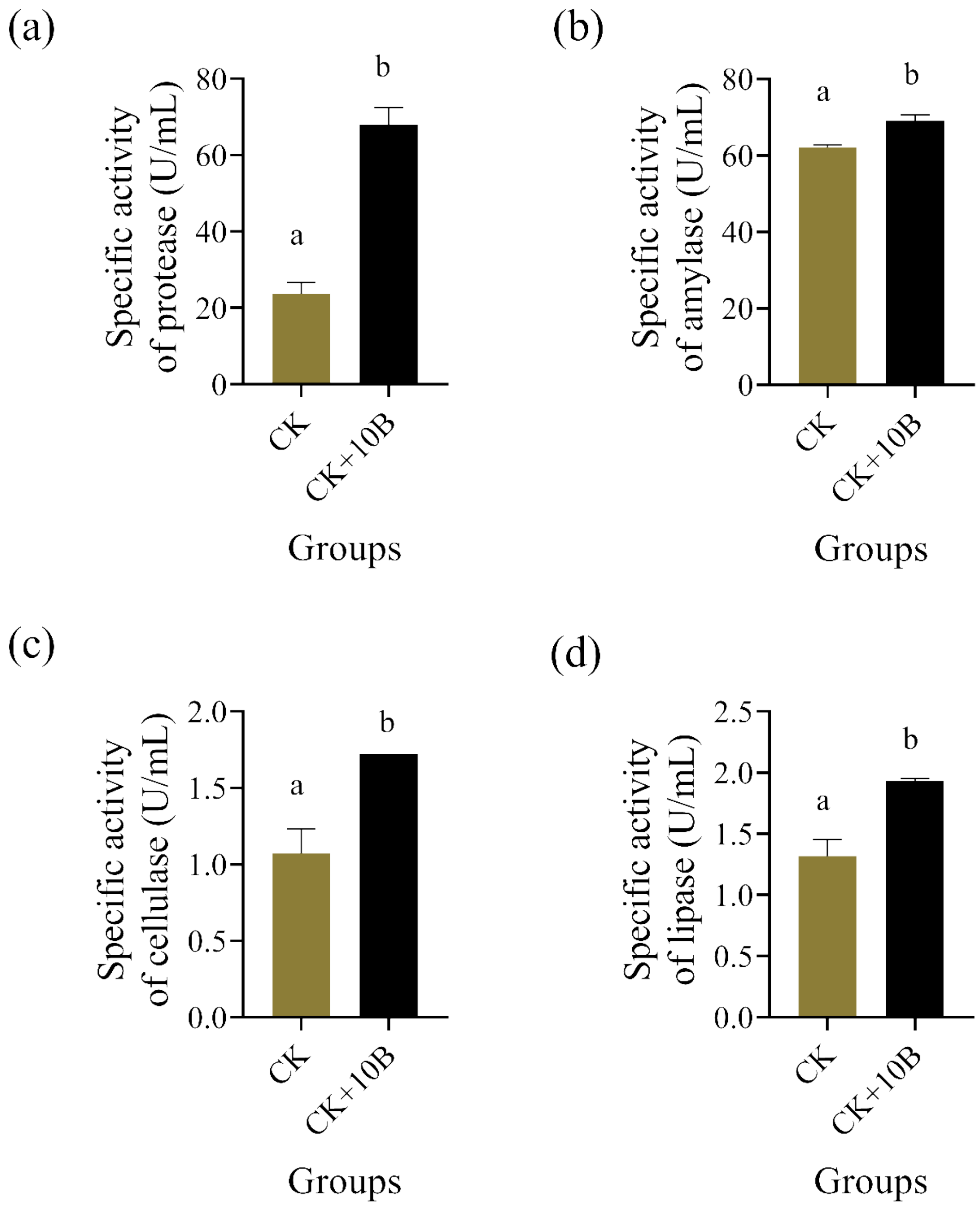

Fig S1. Specific activities (μmole product/mg protein/min) of **(a)** protease, **(b)** amylase, **(c)** cellulase, and **(d)** lipase activity in CK and CK+C10 groups, respectively. CK: natural BSFL fed on non-sterile food waste; CK+10B: natural BSFL fed on non-sterile food waste and *Bacillus velezensis* EEAM 10B. Data are presented as mean ± standard deviation (n = 3). Values with different letters mean significant differences at $p < 0.05$, as determined by Tukey's test.

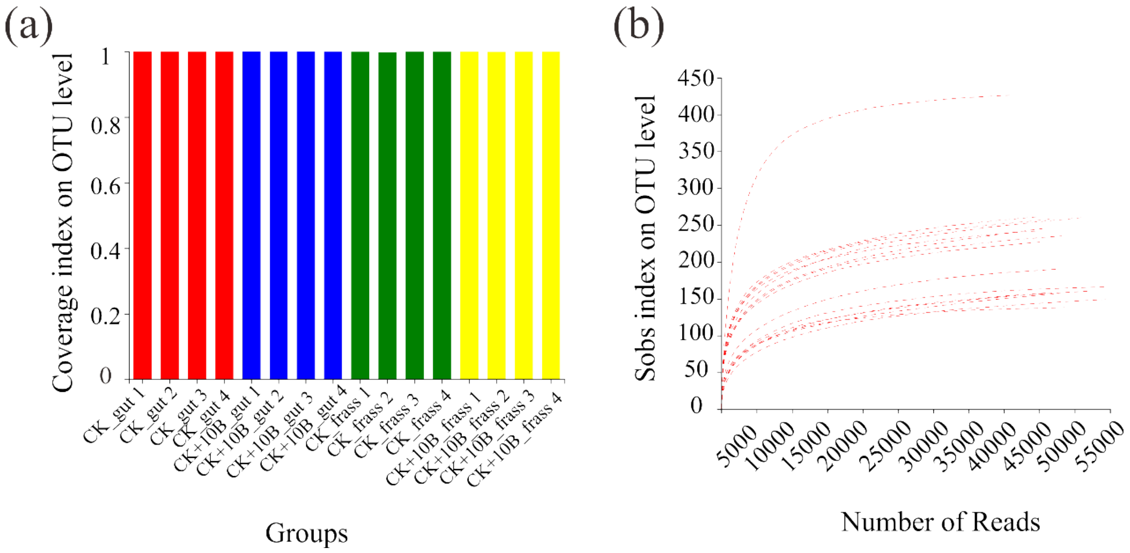

Fig S2. (a) Coverage index calculated based on a cutoff with 97% 16S rRNA gene sequence similarity. **(b)** Rarefaction curve based on OTUs at the cutoff with 97% 16S rRNA sequence similarity in different samples.

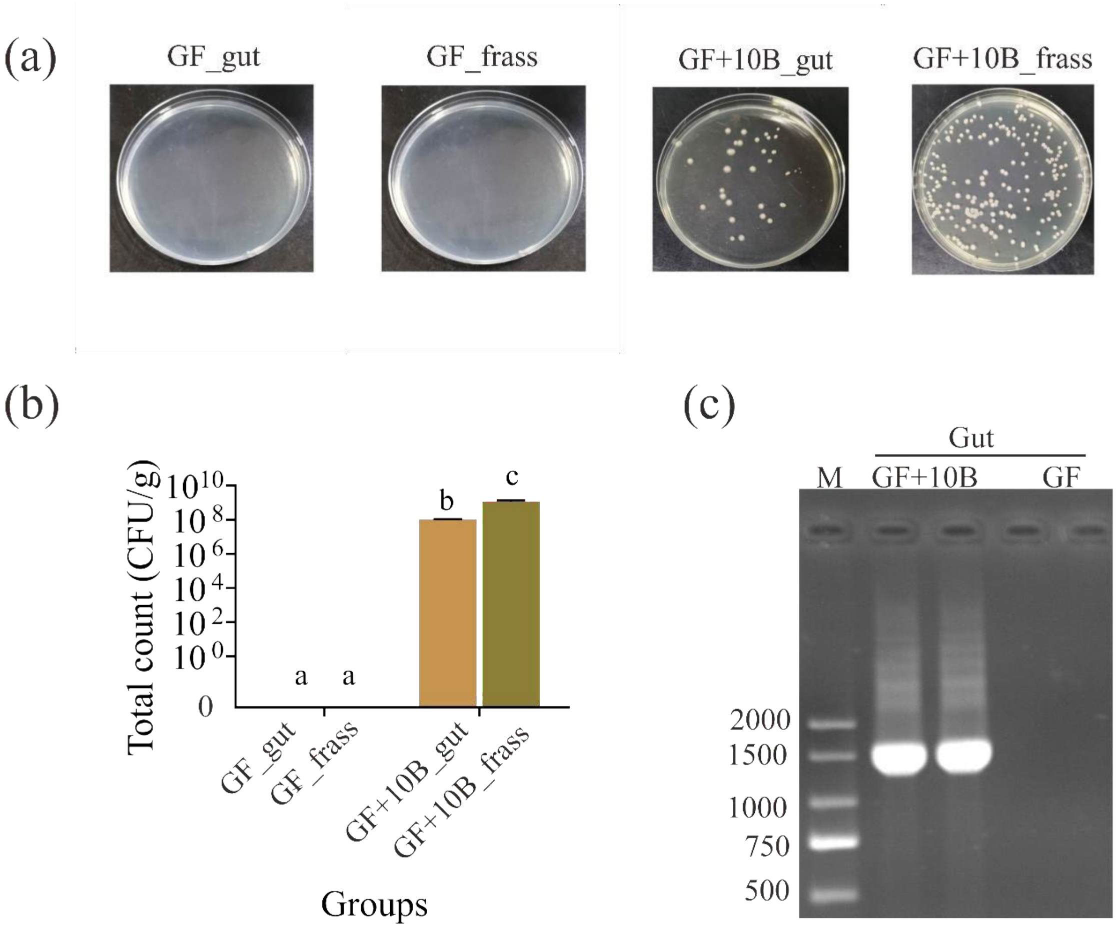

Fig. S3 (a) Germ-free model verified by spreading bacteria at brain heart infusion (BHI)

agar plate. **(b)** CFU counting of gut and frass in GF and GF+10B groups. **(c)** 16S rRNA genes PCR amplified with the gDNA extracted from GF+10B and GF groups, determined by 1.0% agarose gel electrophoresis (5 V/cm, 30 min).

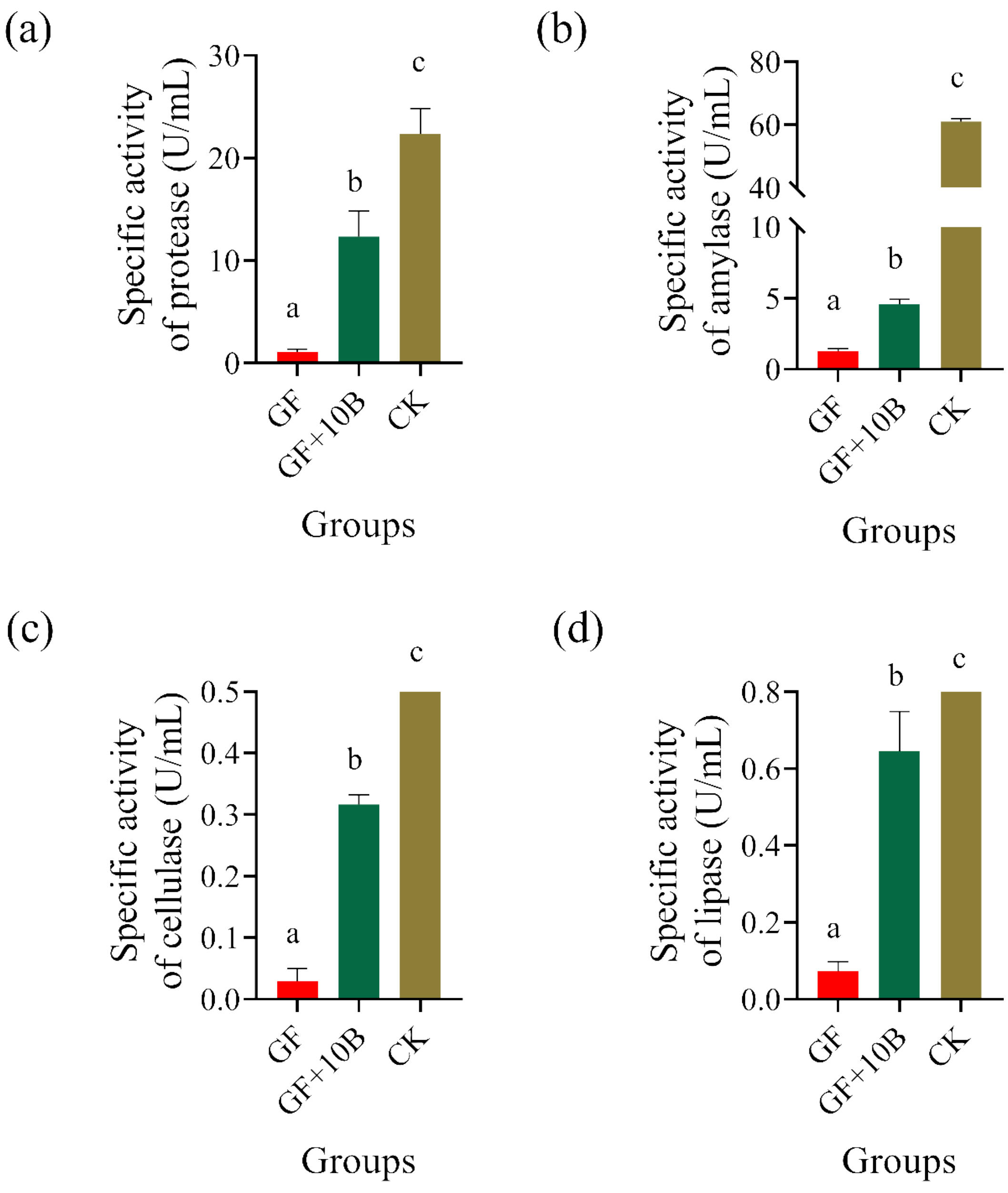

Fig S4. Specific activities (μmole product/mg protein/min) of **(a)** protease, **(b)** amylase, **(c)** cellulase, and **(d)** lipase activity in GF, GF+10B, and CK groups. GF: germ-free BSFL fed on sterile food waste; GF+10B: germ-free BSFL fed on sterile food and

Bacillus velezensis EEAM 10B; CK: natural BSFL fed on sterile food waste. Data are presented as mean ± standard deviation (n = 3). Values with different letters mean significant differences at $p < 0.05$, as determined by Tukey's test.
